# Birth prevalence of selected external structural birth defects at four hospitals in Dar es Salaam, Tanzania, 2011–2012

**DOI:** 10.7189/jogh.05.020411

**Published:** 2015-12

**Authors:** Rogath Saika Kishimba, Rose Mpembeni, Janneth M Mghamba, David Goodman, Diana Valencia

**Affiliations:** 1 Tanzania Ministry of Health and Social Welfare, Tanzania Field Epidemiology and Laboratory Training Programme, Dar es Salaam, Tanzania; 2 Muhimbili University of Health and Allied Sciences (MUHAS), School of Public Health and Social Sciences, Department of Epidemiology and Biostatistics, Dar es Salaam, Tanzania; 3 National Center for Chronic Disease Prevention and Health Promotion, Centers for Disease Control and Prevention, Atlanta, GA, USA

## Abstract

**Background:**

94% of all birth defects (BD) and 95% of deaths due to the BD occur in low and middle income countries, many of which are preventable. In Tanzania, there is currently a paucity of BD data necessary to develop data informed prevention activities.

**Methods:**

A cross-sectional analysis was conducted of deliveries identified with BD in the labor ward registers at four Dar es Salaam hospitals between October, 2011 and February, 2012. The birth prevalence of structural BD, case fatality proportion, and the distribution of structural defects associated deaths within total deaths were calculated.

**Results:**

A total of 28 217 resident births were encountered during the study period. Overall birth prevalence of selected defects was 28.3/10 000 live births. Neural tube defects and indeterminate sex were the most and least common defects at birth (9.9 and 1.1/10 000 live births, respectively). Among stillbirths (66.7%) and deaths that occurred within less than 5 days of an affected live birth (18.5%), neural tube defects were the most frequently associated structural defect.

**Conclusion:**

Structural BD is common and contributes to perinatal mortality in Dar es Salaam. More than half of perinatal deaths encountered among the studied selected external structural BD are associated with neural tube defects, a birth defect with well–established evidence based prevention interventions. By establishing a population–based BD surveillance program, Tanzania would have the information about neural tube defects and other major structural BD needed to develop and monitor prevention activities.

A birth defect is an abnormality of structure or function which originates during intrauterine life and is evident before birth, at birth or manifests later in life [1]. Major structural birth defects are present at birth and they typically have significant medical or surgical consequences. They arise from genetic or environmental causes, some are multifactorial, having both genetic and environmental causes, and for others the causes are still unknown [1].

Birth defects may be mild or severe. Major structural birth defects include congenital heart disease (CHD), neural tube defects (NTDs), orofacial clefts, and limb reduction defects; these defects are considered severe, having adverse effects on the well-being and survival of children born with those anomalies [1,2]. Almost all birth defects (94%) and deaths due to the birth defects among children (95%) occur in low and middle income countries [2]. The global mortality associated with birth defects, as reported by the March of Dimes (MOD) is estimated at 3.3 million children under age five years dying from serious birth defects. Of those children who survive, it is estimated that 3.2 million may be disabled for life, without appropriate care. Birth defects exact a severe human and economic toll on those affected, their families and their communities [2,3].

In Tanzania, it is estimated that the prevalence of birth defects is 60.5 per 1000 live births [2]. Studies done at Muhimbili National Hospital neonatal unit in Dar es Salaam have shown a birth defects prevalence of 33 per 1000 live births, and a prevalence of 3.0 per 1000 live births for NTDs [4,5]. Eight percent of the overall neonatal mortality was attributed to birth defects [5]. There remains a paucity of data in Tanzania on birth defects. This is due to constrained diagnostic capabilities, lack of awareness of available services, and the absence of a birth defects surveillance system and registry. The absence of routine, reliable and systematically collected data prevents the development of information necessary to develop, monitor, and evaluate prevention strategies. This study establishes the magnitude of selected major structural defects in Dar es Salaam, Tanzania and characterizes the burden, providing evidence for the usefulness of a birth defects surveillance system, including the development of data informed birth defects prevention activities.

## METHODS

This study included all newborns delivered from October, 2011 through February, 2012 in Dar es Salaam from Muhimbili National Hospital (MNH) and all three Municipal hospitals (Temeke, Mwananyamala and Amana). Ninety percent of births to Dar es Salaam residents occur in a health facility, and 72% of Dar es Salaam residents deliver at Muhimbili National Hospital or at one of the three municipal hospitals [6,7]. These hospitals are the biggest public hospitals in Dar es Salaam, serving populations with diverse ethnic and demographic characteristics as well as health related behaviors. None of the municipal hospitals are referral hospitals for mothers with a prenatal diagnosis of a fetus with birth defects as this is not in our standard Reproductive and Child Health Antenatal Recommendation, and termination of pregnancies for fetal anomalies are not allowed.

A case was defined as any live birth or stillbirth identified at delivery with a selected external major birth defect in any of the four hospitals during the study period. Study data came from a review and abstraction of labor ward registers during the study period (October 2011 to February 2012). Data were abstracted daily by trained midwives. Labor ward admission and discharge procedures were similar in all study sites. Every pregnant woman who was in labor pain was admitted and registered. Her particulars like name, age, place of residence, gravity and parity are recorded. After giving birth, a complete clinical evaluation of the infant is done by the Medical Officer and if he/she had any medical problem including birth defects, he/she is sent to the neonatal unit for further care and treatment. If he/she has no problem, the baby is given to his/her mother, which will stay at least 24 hours before being discharged.

The international classification of diseases version 10 (ICD10) was used to code selected external major structural defects [8]. The selected structural defects were further classified as having only one major birth defect (isolated), having more than one major birth defect (multiple) or occurring as part of a genetic or chromosomal condition (syndrome). All newborns delivered during the study period to Dar es Salaam resident mothers (women who lived in Dar es Salaam for the 6 months prior to delivery) were included in this study. A standard data collection form was developed and used to collect maternal, paternal, and newborn demographic data. The form included collecting the patient registration number to avoid duplication of cases, along with the name of the delivery hospital, date of birth, sex of the newborn, and type of birth defect.

Data were entered, cleaned, and analyzed using Epi Info Version 3.5.1. (Centers for Disease Control and Prevention, Atlanta, GA, USA). The birth prevalence of selected major structural defects was calculated by dividing the total number of newborns (live and stillbirths) with selected structural defects (central nervous system defects, orofacial clefts, congenital malformations of the genital organs, musculoskeletal defects, and chromosomal abnormalities) delivered during the study period (Numerator) by the total number of live births delivered at participating hospitals during the same time period (Denominator) [9]. The distribution of deaths within defect types was calculated by dividing the number of deaths associated with a specific defect by the total number of deliveries affected by that specific defect (live births + still births), multiplied by 100. This was calculated separately for live births that survived less than 5 days and stillbirths. The distribution of selected structural defects associated deaths within total deaths was calculated by dividing the number of defect specific deaths by the total number of defect associated deaths. This was calculated separately for live births that survived less than 5 days and stillbirths.

The protocol for this study was approved by the Internal Review Board of Muhimbili University of Health and Allied Sciences (MU/PGS/SAEC/Vol.VI/2011) and Muhimbili National Hospital (No. 150 2011/2012). Names of respondents were not recorded in the data collection form and measures were taken to ensure confidentiality and security of the information collected.

## RESULTS

During the study period, a total of 28 217 deliveries occurred in the four participating hospitals of which 27 230 were live births. Seventy seven newborns (28.3 per 10 000 live births) had one of the selected external major structural defects. Of the 77 newborns with selected structural defects, 38 (49.3%) were males, 36 (46.8%) females and 3 (3.9%) had undetermined sex (**Table 1**). When considering all deliveries in the denominator, the birth prevalence of selected structural defects is similar to when only live births are included in the denominator for calculating birth prevalence (27.3 per 10 000 total births). Males and females had a similar overall birth prevalence of selected structural defects, though NTDs were more common among females, and isolated hydrocephalus and orofacial defects were more common among males. Neural tube defects had the highest overall birth prevalence (9.9/10 000 live births) among the selected external major structural defects. Indeterminate sex defects and chromosomal abnormalities had the lowest overall birth prevalence among the selected structural defects, respectively (1.0/10 000 live births and 1.8 /10 000 live births).

**Table 1:** Birth prevalence of selected major structural birth defects, by sex, Muhimbili National Hospital and three Municipal hospitals in Dar es Salaam, Tanzania, 2011–2012

Birth defect	ICD 10	Live births and fetuses with birth defects (Count (n), prevalence per 10 000 live births (P); 95% Confidence interval (CI) (95%)			
**Male**	**Female**	**Undetermined**	**Total**
N = 13 550	N = 13 677	N = 3	N = 27 230
**CNS defects**	–	n = 18 (*P* = 13.3)	n = 21 (15.4)	–	n = 39 (*P* = 14.3; 95% CI = 10.2–19.6)
**Neural tube defects:**	–	n = 10 (*P* = 7.4)	n = 17 (*P* = 12.4)	–	n = 27 (*P* = 9.9; 95% CI = 6.5–14.4)
Anencephaly	Q00.0	n = 5 (*P* = 3.7)	n = 9 (*P* = 6.6)	–	n = 14 (*P* = 5.1;95% CI = 2.8**–**8.6)
Spina bifida	Q05	n = 5 (*P* = 3.7)	n = 5 (*P* = 3.7)	–	n = 10 (*P* = 3.7; 95% CI = 1.8–6.6)
Encephalocele	Q01	n = 0 (*P* = 0.0)	n = 3 (*P* = 2.2)	–	n = 3 (*P* = 1.1; 95% CI = 0.2–3.2**)**
**Isolated hydrocephalus**	Q03	n = 8 (*P* = 5.9)	n = 4 (*P* = 2.9)	–	n = 12 (*P* = 4.4; 95% CI = 2.3–7.7)
**Orofacial clefts:**	–	n = 7 (*P* = 5.2)	n = 4 (*P* = 2.9)	–	n = 11 (*P* = 4.0; 95% CI = 2.0–7.2)
Cleft palate	Q35	n = 2 (*P* = 1.5)	n = 1 (*P* = 0.7)	–	n = 3 (*P* = 1.1; 95% CI = 0.2–3.2)
Cleft lip	Q36	n = 3 (*P* = 2.2)	n = 1 (*P* = 0.7)	–	n = 4 (*P* = 1.5; 95% CI = 0.4–3.8)
Cleft palate with cleft lip	Q37	n = 2 (*P* = 1.5)	n = 2 (*P* = 1.5)	–	n = 4 (*P* = 1.5; 95% CI = 0.4**–**3.8)
**Indeterminate sex**	Q56	–	–	n = 3 (*P* = 1.5)	n = 3 (*P* = 1.1;95% CI = 0.2–3.2)
**Musculoskeletal defects:**		n = 10 (*P* = 7.4)	n = 9 (*P* = 6.6)	–	n = 19 (*P* = 7.0;95% CI = 4.2–10.9)
Talipes equinovarus	Q66.0	n = 8 (*P* = 5.9)	n = 7 (*P* = 5.1)	–	n = 15 (*P* = 5.5; 95% CI = 3.1–9.1)
Reduction defects of upper and lower limbs	Q71 & Q72	n = 2 (*P* = 1.5)	n = 2 (*P* = 1.5)	–	n = 4 (*P* = 1.5; 95% CI = 0.4–3.8)
**Chromosomal abnormalities:**	–	n = 3 (*P* = 2.2)	n = 2 (*P* = 1.5)	–	n = 5 (*P* = 1.8; 95% CI = 0.6–4.3)
Down syndrome	Q90.9	n = 1 (*P* = 0.7)	n = 2 (*P* = 1.5)	–	n = 3 (*P* = 1.1; 95% CI = 0.2–3.2)
Edward syndrome	Q91.3	n = 2 (*P* = 1.5)	–	–	n = 2 (*P* = 0.7; 95% CI = 0.1–2.7)
**Total**	–	n = 38 (*P* = 28.0)	n = 36 (*P* = 26.3)	n = 3 (*P* = 1.0)	n = 77 (*P* = 28.3; 95% CI = 22.3–35.3)

The majority of selected external structural defects were isolated (74%), while19.5% had multiple anomalies and 6.5% had syndromes. Overall, 76.6% of deliveries (live births + stillbirths) with selected external structural defects survived at least 5 days, with 15.6% dying prior to delivery (stillbirth), and 7.8% dying within 5 days of delivery (**Table 2**). Neural tube defects had the highest defect–specific mortality among live births and total deliveries (live births + stillbirths) and represented the majority of stillbirths and under 5–day deaths among selected structural defect–affected pregnancies.

**Table 2:** Counts, distribution of deaths within specific defects, and distribution of deaths within live births and stillbirths, National hospital and three Municipal hospitals in Dar es Salaam, Tanzania, 2011–2012

Defect	Survived ≥5 days	Survived <5 days			Stillbirth			Total
**No.**	**No.**	**% within specific defects**	**% within live births**	**No.**	**% within specific defects**	**% within stillbirths**	**No.**
Neural tube defects (anencephaly, spina bifida, encephalocele)	14	5	18.5	83.3	8	29.6	66.7	27
Isolated hydrocephalus	9	1	8.3	16.7	2	16.7	16.7	12
Orofacial clefts (palate, lip, palate with lip)	10	0	0	0	1	9.1	8.3	11
Indeterminate sex	2	0	0	0	1	33.3	8.3	3
Musculoskeletal defects (talipes equinovarus, reduction of upper and lower limbs)	19	0	0	0	0	0	0	19
Chromosomal abnormalities (Down syndrome, Edward syndrome)	5	0	0	0	0	0	0	5
Total	59	6	7.8	100	12	15.6	100	77

## DISCUSSION

In the diverse birth population of Dar es Salaam, we observed that NTDs, one of the selected external structural defects with the greatest opportunity for prevention, were the most prevalent structural defect followed by musculoskeletal defects. This finding is in contrast to other studies conducted in different parts of the world – Uganda, Nigeria, South Africa and Israel– where musculoskeletal defects were the most common birth defects [10–14]. This may be due to our study’s focus on just two major birth defects of the musculoskeletal system. Our observation that NTD birth prevalence was slightly higher in female newborns than males is similar to a study done in Iran whereby they observed two thirds of NTD–affected newborns were female [15]. However findings from studies conducted in Nigeria were in contrast to our observation [16,17]. The difference may relate to differences in the distribution of specific NTD types between the populations, as we observed the difference was driven by two of the three major NTD types. In our study, the birth prevalence of anencephaly was higher than that of spina bifida; this finding was comparable to studies done in Yaoundé Cameron and Texas, United States which observed a higher prevalence of anencephaly than spina bifida [18,19]. Neural tube defects prevalence was comparable to the prevalence reported by India to the International Clearinghouse for Birth Defects Surveillance and Research (ICBDSR) [20].

We did not identify a significant birth defect prevalence difference when considering only live births and total deliveries as denominators. Isolated birth defects were more common than multiple and syndrome birth defects, a finding similar to a prospective neurosurgical observational study done in Nigeria to assess central nervous system congenital anomalies [16]. Among NTD–affected newborns the percentage who were stillborn and who were alive for less than 5–days was higher than for other selected external structural defects. This likely reflects that NTDs are the most serious and fatal birth defects compared to the other defects included in this study as has been previously reported [21,22]. A study done in the United Kingdom among male radiation workers at the Sellafield nuclear processing plant showed that risk of stillbirth among their offspring was highest among NTD–affected fetuses [23]. In a hospital based epidemiological descriptive study done in Iran that reviewed live births and stillbirths for a period of 4.5 years, showed a prevalence rate of NTDs among stillbirths being more than twice that among live births [15].

Evidence shows that preconceptional use of folic acid helps prevent NTDs, and many countries are implementing mandatory fortification of folic acid in cereals [24–28]. In the United States folic acid fortification resulted in an approximate 19% decrease in the incidence of NTDs [24]. Folic acid supplementation, through the consumption of vitamins, is an alternative approach to fortification that has been shown to reduce the primary incidence of NTD by 62% and recurrence of NTD by 70% [28]. In developed countries, folic acid supplementation policy faces significant challenges from unplanned pregnancies, lack of easy access to a functioning health system and effective local social marketing interventions [29–31]. In developing countries like Tanzania, folic acid supplementation policy will be difficult to implement given that these countries have high levels of poverty, poor health care infrastructure, and high rates of unplanned pregnancies compared to their counterparts [32–34]. Mandatory folic acid fortification policy is an option for developing countries to consider, which overcomes some of the challenges of supplementation. Tanzania is currently implementing a mandatory large scale wheat flour fortification policy. Having a birth defects surveillance system in place will help facilitate monitor this prevention strategy, and will help identify populations at risk in need of targeted interventions.

Interpretation of these study findings need to be considered in relation to several strengths and weaknesses. One of the major strengths of this study is that all births were thoroughly evaluated during the study period by clinical professionals. This made it possible to capture birth defects among stillbirths or births that survived less than 5 days, which would not have been possible if our study ascertained birth defects by either retrospective review of data from neonatal units or collecting data from either neonatal admission units or birth defects clinics alone. These alternative approaches would have resulted in underestimating the burden of structural birth defects included in this study. An additional strength of this study is the involvement of multiple public hospitals, representing the majority of deliveries to Dar es Salaam residents and covering people with a diversity of demographic characteristics and health related behaviors.

This study was limited by the duration of follow–up. Newborns with structural birth defects were followed up for only 5–days, and so the contribution of the selected structural defects to early mortality is a likely underestimate, though there is no evidence that the distribution of selected defects among deaths would differ at 2–weeks or 1–month after delivery. Also only selected major external birth defects were included during the study period to facilitate identification and avoid under reporting. Internal major birth defects were not included due to limitations in technology to diagnose them. Another limitation of this study was that it only included hospital deliveries, which could have either led to underestimation or overestimation of birth prevalence; however, 90.2% of births to Dar es Salaam residents occur in health facilities [6]. Our study was facility–based, and so generalizability of the findings are limited to births in the study facilities; however, deliveries at these facilities represent 72% of all deliveries in Dar es Salaam [7].

## CONCLUSION

This study demonstrates that structural external major birth defects are frequent in clinical practice in Dar es Salaam. NTDs were the most common occurring, followed by musculoskeletal defects and orofacial clefts. The majority of stillbirths with selected external structural defects were associated with a neural tube defect which has a well–established evidence based prevention interventions. We can therefore lower perinatal mortality through preventing neural tube defects particularly spina bifida and anencephaly.

By establishing a population based birth defects surveillance program, which can provide accurate and reliable estimates of the prevalence and risk factors for NTDs and other major birth defects, Tanzania will have the information necessary for the effective development and monitoring of birth defects prevention activities, including folic acid fortification.
